# A call to action: impact of climate change on TB and recovery after TB

**DOI:** 10.5588/ijtldopen.25.0734

**Published:** 2026-03-13

**Authors:** A. Mabhula, G. Hoddinott, H. Sohn, C.Y. Wright, D. Evans

**Affiliations:** 1 Health Economics and Epidemiology Research Office, School of Clinical Medicine, Faculty of Health Sciences, University of the Witwatersrand, Johannesburg, South Africa;; 2 Desmond Tutu TB Centre, Department of Paediatrics and Child Health, Faculty of Medicine and Health Sciences, Stellenbosch University, Cape Town, South Africa;; 3 School of Public Health, Faculty of Medicine and Health, The University of Sydney, Sydney, NSW, Australia;; 4 The University of Sydney Infectious Diseases Institute (Sydney ID), Sydney, NSW, Australia;; 5 Department of Preventive Medicine, Seoul National University College of Medicine, Seoul, South Korea;; 6 Department of Human Systems Medicine, Seoul National University College of Medicine, Seoul, South Korea;; 7 Institute of Health Policy and Management, Seoul National University Medical Research Center, Seoul, South Korea;; 8 Climate Change and Health Research Programme, Environment and Health Research Unit, South African Medical Research Council, Pretoria, South Africa;; 9 Department of Geography, Geoinformatics and Meteorology, University of Pretoria, Pretoria, South Africa.

**Keywords:** tuberculosis, climate change, post-TB recovery, TB risk factors, climate-sensitive policies

Dear Editor,

TB morbidity and mortality remain high, disproportionately affecting low- and middle-income countries (LMICs).^1^ Environmental and climate-related factors, including inequality, poverty, overcrowding, inadequate ventilation and housing, and food and water insecurity, directly and indirectly increase susceptibility to TB.^2^ These factors not only worsen the clinical and financial burden among those affected but also contribute to increased transmission. These problems are further exacerbated in LMICs due to highly strained health systems, affecting the cost and quality of TB care. With growing recognition of asymptomatic TB, post-TB lung disease (PTLD), and other long-term TB-related sequelae, the health and socio-economic consequences associated with TB will likely become greater with increased global climate change.

In 2020, an estimated 155 million people globally were living after completing TB treatment (TB survivors)^3^ and face elevated risks of non-communicable diseases. TB survivors face higher risks of recurrent TB and premature death than the general population,^4–6^ ongoing exposure to structural risk factors that affect them and their networks, and the potential to transmit TB during subsequent asymptomatic episodes. Further challenges often arise due to stigma and unemployment, leading to financial hardships, reinforcing a cycle of poverty and poor health.^7^ PTLD, which affects an estimated 60% (range 28%–87%) of individuals cured of TB,^8^ is defined by a history of pulmonary or pleural TB without active disease at assessment, evidence of pulmonary abnormalities in at least two of three domains – lung function, respiratory symptoms, or chest imaging – and findings attributable, at least in part, to prior TB disease.^9^ People with PTLD often experience significant, potentially lifelong physical, social, psychological, and economic impacts resulting in reduced quality of life, greater vulnerability to further respiratory illnesses, and the need for ongoing health care.^4,5,10^ These challenges highlight the need for integration of post-TB treatment follow-up and comprehensive TB intervention programmes to prevent TB-associated disability^11^ and premature death across the entire TB care continuum.

Climate change is increasingly being recognised as a threat to infectious disease control and prevention mechanisms.^12^ Subsequently, the WHO has identified TB as a climate-sensitive disease and published a framework highlighting the risk of climate change on worsening the TB epidemic due to its effects on TB risk factors.^13^ Unlike infectious diseases whose patterns of vector transmission are directly impacted by rising temperatures and shifting rainfall and thereby altering the risk of disease in certain geographical areas,^14^ the causal link and impact of climate change on TB are generally indirect in nature.^15^ Consequently, the effects of climate change on TB are often overlooked in broader climate and health discussions, leaving countries without clear guidance on how to respond. Existing analytic frameworks focus on transmission risk, disease progression, and TB treatment and outcomes, but do not account for how climate change may increase the risk or severity of TB-associated chronic respiratory conditions, hinder TB recovery, or increase the risk of TB recurrence.^12,13^ Equally important, these frameworks overlook the role of rehabilitation therapy in recovery and ignore how climate change may drive a greater need for rehabilitation and mental health support while also disrupting care, making it harder for survivors to receive the help they need.^16^ In response, we propose a conceptual framework that outlines potential impacts of climate change on enduring sequelae post-TB treatment completion (Figure).

**Figure.**
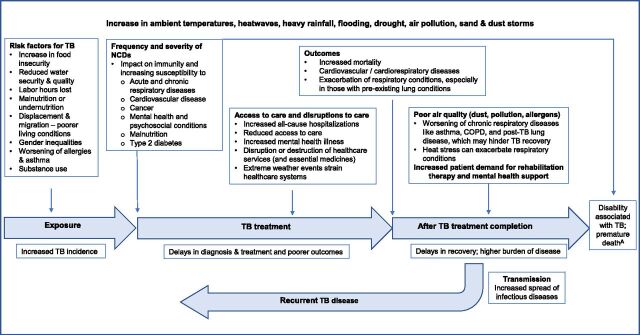
The impact of climate change on the enduring impacts of TB. ^A^People with disabilities face greater risks of injury, illness, and mortality during extreme weather conditions and experience more severe disruptions to their care. COPD = chronic obstructive pulmonary disease; NCD = non-communicable disease.

Climate change, including changes in temperature, humidity, precipitation, and the frequency of extreme weather events (Figure), worsens the social determinants of TB, such as poverty, displacement, food insecurity, and limited access to health care, thereby influencing the distribution and burden of disease. The impact of these events will be more pronounced in high-burden countries, including LMICs, causing further strain on vulnerable health systems. Droughts and flooding in impoverished communities can lead to population displacement or climate-induced migration and disruption of health services, and impact water and food security.^15^ Migration of vulnerable populations often leads to poor living conditions, overcrowding, inadequate ventilation, and overburdened health systems, which can accelerate TB transmission. Moreover, malnutrition, often exacerbated by droughts, impairs immune function, thus increasing susceptibility to TB infection and latent TB reactivation. The co-occurrence of climate change–related events, including flooding and droughts, can create overlapping vulnerabilities that amplify the burden of TB.

Climate change–related environmental factors such as air pollution, allergens, and heat stress have also been shown to negatively affect lung health and cause inflammation, which further exacerbates chronic respiratory conditions, including PTLD. Rising rates of TB-related mortality, morbidity, and disability, exacerbated by climate change, will contribute to the total burden of TB disease. Further studies are needed to investigate and understand the extent of these pathways and to determine where global TB efforts should be intensified.

By creating awareness of the impact of climate change on TB, we can advocate for change. Interventions at the macro-, meso-, and micro-levels can be adopted to create climate-resilient health care systems. At the macro level, especially in high-TB-burden countries, governments should embed climate resilience into health systems and national TB strategies and adopt climate-sensitive policies that enable multisectoral action, such as water-security planning and urban greening. TB surveillance systems should integrate monitoring of environmental factors and social determinants of health to better understand the context in which TB cases develop and spread. At the meso level, local and regional policymakers and stakeholders should collaborate across sectors, for example, environment, health, housing, and spatial planning, to develop interventions such as well-ventilated clinical and hospital spaces, shaded outdoor waiting areas, heat-stress monitoring, and other climate-responsive measures. Furthermore, disaster risk management plans should incorporate continuity of TB health care services during climate-related emergencies, such as flooding. Finally, at the micro-level, community engagement is essential. Researchers suggest that TB prevention could reduce climate-change impacts.^17^ Education and awareness campaigns should empower people to understand the links between climate change, TB transmission, and post-TB recovery. People should be encouraged to adopt healthy lifestyles, be educated on weather preparedness, and the health risks associated with heat. Although modest, patient-centred approaches such as decentralised service delivery may help to reduce the health care system’s environmental footprint by lowering energy use in clinics and reducing the need for patient transportation.

Evidence related to the impact of climate change on TB and post-treatment recovery is limited. We need novel methodologies, big data, and multidisciplinary approaches that combine epidemiological, climate modelling, geospatial, and mixed-method survivor experience studies to generate such evidence. Similarly, investments in standardised data collection systems will be needed to synchronise, synthesise, and analyse longitudinal data. Cross-sector collaborations between health and other relevant sectors are crucial to minimising disruptions to essential TB services and maximising or strengthening climate change resilience. Without integrating climate resilience strategies into TB control programmes, we risk undermining the decades of progress in TB prevention and elimination. Climate inaction will mostly impact those already severely affected by TB, reinforcing the burden of disease and undoing much of the potential benefit of biomedical and health system innovations.
